# Upper Limb Neurodynamic Test 1 on Healthy Individuals: Intra- and Intersession Reliability of the Angle between Pain Onset and Submaximal Pain

**DOI:** 10.1155/2016/9607262

**Published:** 2016-09-22

**Authors:** Diego Leoni, Davide Storer, Roberto Gatti, Michele Egloff, Marco Barbero

**Affiliations:** ^1^Rehabilitation Research Laboratory 2rLab, Department of Business Economics, Health and Social Care, University of Applied Sciences and Arts of Southern Switzerland, SUPSI, Manno, Switzerland; ^2^Rehabilitation Department, San Raffaele Hospital, Milan, Italy; ^3^Humanitas University and Humanitas Clinical and Research Center, Rozzano, Italy

## Abstract

Assessment of nerve trunk mechanosensitivity using the upper limb neurodynamic test 1 (ULNT1) often includes measurement of the angle of occurrence in the range of pain onset (PO) and submaximal pain (SP). A measurement that better fits the idea of mechanosensitivity could be the angle between PO and SP (AbOS). This study investigated the intra- and intersession reliability of AbOS, PO, and SP during the ULNT1. Forty-four healthy volunteers underwent three ULNT1 to the point of PO and SP, twice in the first session and once in the second. AbOS, PO, and SP angles of occurrence reliability were examined using the Intraclass Correlation Coefficient (ICC 3,1) and Bland-Altman plots. The intra- and intersession ICC values for AbOS were 0.71 (95% CI: 0.47; 0.85) and 0.79 (95% CI: 0.60; 0.89), respectively. The intra- and intersession mean difference and 95% limits of agreement (±1.96 SD) in the Bland-Altman plots were 2.3° (−18.3°; 23.1°) and 2.8° (−14.7°; 20.4°), respectively. The intra- and intersession reliability of the AbOS during the ULNT1 in healthy individuals is high and higher than the reliability of PO and SP angles of occurrence. The AbOS could be a preferable variable in the assessment of neural mechanosensitivity.

## 1. Introduction

Neurodynamic tests are common procedures used by physiotherapists treating patients with disorders involving musculoskeletal pain to assess nerve mechanosensitivity and test the involvement of neural structures as a possible source of pain [1–8]. They consist of a precise sequence of movements that elongate the nerve bedding and increase the pressure in and around peripheral nerves and nerve trunks [9]. Mechanosensitivity in neurodynamics is defined as the sensitivity to mechanical stress applied to peripheral neural structures [10, 11]. It can be increased as a result of different causes such as chemical and physical damage to peripheral nociceptive afferents [12], development of abnormal impulse-generating sites [13], lowered threshold of mechanoreceptors [14], emotional stress [15–17], and sensitization of the central nervous system [13]. The upper limb neurodynamic test 1 (ULNT1) is the test most commonly used in the assessment of upper quadrant disorders [2, 6–8, 18].

The application of mechanical stress to peripheral nerves and nerve trunks by the ULNT1 can provoke pain and other sensory responses (e.g. burning, stretching, and tingling) [4, 19]. However, it must be considered that pain and sensory responses during ULNT1 have been documented both in musculoskeletal pain patients [3–5, 20, 21] and in healthy people [22–24] and that it is not always easy to discriminate whether responsiveness to the test is normal or increased [13, 17, 25, 26]. This is the reason why, as reported in a recent literature review on UNLT validity [5], two main conditions must be met before considering a positive neurodynamic test: these are provocation of patient symptoms and symptom change (increase or decrease) in response to structural differentiation [1, 5, 13]. Other common variables taken into account for mechanosensitivity assessment are different responses between the symptomatic and the asymptomatic side, joint angle degree at the moment of pain onset (PO), and submaximal pain (SP) and resistance to movement during the test [5, 9, 23, 24, 27].

Almost all the criteria for mechanosensitivity assessment rely on subjective findings as referred by patients (pain occurrence) or determined by the examiners (resistance to movement). The only proposed method to quantify mechanosensitivity is joint angle measurement (e.g. “elbow extension” for the ULNT1) at the moment of PO and SP [9, 23, 24]. All studies on this issue reported “high to very high” intratester/intrasession reliability in detecting the angles of PO and SP occurrence, both in the laboratory and in clinical setting, making this measure an acceptable variable in the interpretation of ULNT1 [9]. Unfortunately, no data on the intratester and intersession reliability are available, thereby actually limiting the applicability of this measurement to the evaluation of changes in mechanosensitivity over time. For the complexity of the ULNT1 procedure, even a small difference in subject positioning between sessions may affect the angle of PO and SP occurrence, thus also potentially affecting the reliability of this measurement. However, it could be reasoned that even if PO and SP in two different sessions occurred at different points in the range, the angle between PO and SP could be a more stable measurement. If so, an alternative method to clinically quantify mechanosensitivity during ULNT1 might be measurement of the angle between the occurrence of PO and SP (AbOS). Furthermore, it could be reasoned that AbOS (measuring the irritability of nerve trunks during the application of a progressive mechanical stress) is a more valid measurement of nerve mechanosensitivity. In fact, PO and SP angles of occurrence simply tell us at what point of the range of motion the pain starts and when it becomes intolerable, while the AbOS could indicate how early the pain becomes intolerable from the moment of its appearance; this is more in accordance with the concept of mechanosensitivity.

This study on healthy participants aimed to investigate the intra- and intersession reliability of AbOS and to explore whether it was higher than the reliability of PO and SP angles of occurrence.

## 2. Materials and Methods

### 2.1. Study Design

All the experimental sessions were conducted between April and June 2013 in the Laboratories of Movement Analysis of the San Raffaele Hospital (Milano, Italy).

The procedures were conducted according to the Declaration of Helsinki. The study respected the* Ethical Guidelines for Pain Research in Humans*. All volunteers signed an informed consent form prior to the study. The proposed methodology was developed according to the* Guidelines for Reporting Reliability and Agreement Studies* [28].

### 2.2. Participants

A convenience sample of 44 healthy participants (17 female, 27 male; mean age 21.8 ± 2.3 y, mean high 174.4 ± 8.7 cm, mean weight 65.3 ± 8.6 kg) was recruited from the students of the Bachelor in Physiotherapy Programme of the Vita-Salute San Raffaele Hospital University (Milano, Italy).

The inclusion criteria were nonpainful full active range of motion (ROM) for bilateral shoulders, elbows, wrists, hands, and cervical spine. Exclusion criteria were current or recent (at least three consecutive days in the past six months) neck or upper extremity pain, nervous system disorders, diabetes mellitus, upper extremities, breast or cervical spine surgery, drug or alcohol abuse, and radiation therapy or chemotherapy in the past year. The sample size was set according to suggestions by Giraudeau and Mary [29] for the reliability studies and it also met the recommendations of other authors [30, 31]. The operator performing the ULNT1 was a graduating student of the Bachelor of Physiotherapy trained to properly manage the experimental procedures.

### 2.3. Equipment

**Figure 1:**
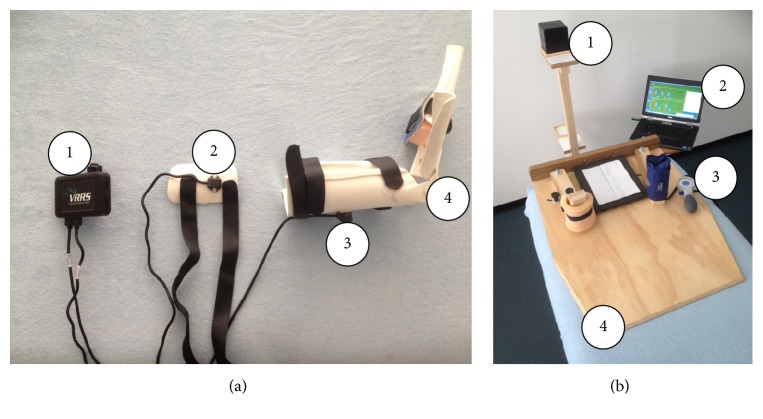
Equipment. (a) (1) Polemus G4 wi-fi hub, (2-3) G4 sensors for arm and forearm respectively, (4) wrist and hand splint. (b) (1) G4 antenna, (2) goniometer software, (3) sphyngomanometer and pressure unit, and (4) wooden positioning device.

**Figure 2:**
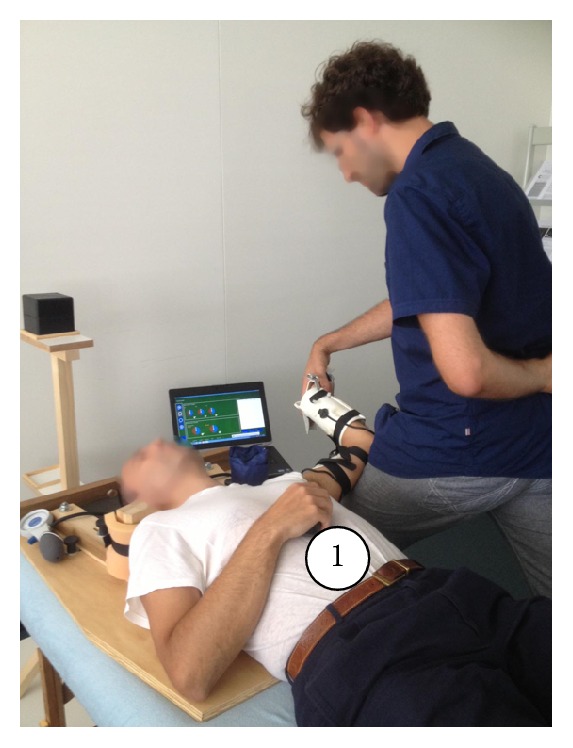
ULNT1 starting position. (1) Switch device held by the participant.

An electromagnetic tracking device (G4, Polhemus Inc., Colchester, Vermont, USA) in combination with the custom-made goniometer software provided real-time visual feedback of gleno-humeral movement degrees (Figures 1(a) and 1(b)), which allowed the operator to perform the ULNT1 as accurately as possible. The device, including six-degrees-of-freedom sensors, was capable of detecting degrees of gleno-humeral abduction, horizontal adduction, and external rotation movement. The system had been used and shown to be accurate to within ±0.2° [32, 33]. A wooden frame for the shoulder girdle, a splint for the wrist and hand, a pressure unit, and a wooden set square were finally used to standardize the starting position (Figure 1(b)). A wireless switch (Figure 2) was used to record the angles at which PO and SP occurred. A visual analogic scale (VAS) was used to measure pain intensity at the moment of SP.

### 2.4. Procedures

**Figure 3:**
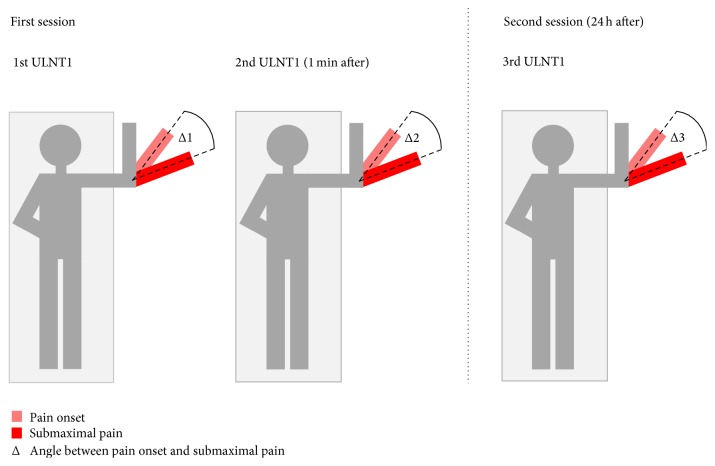
Experimental procedure scheme. The angles of occurrence in range of pain onset and submaximal pain during elbow extension were recorded in each ULNT1; then the angle between PO and SP was calculated for each ULNT1 execution.

*ULNT1 Procedure*. ULNT1 was performed according to the description of Butler and Matheson [13], except for the wrist and fingers extension which became the first movement component, since the splint had to be placed before the participants laid in a supine position. ULNT1 was performed until PO and SP thrice on each participant: twice in the first session (with a time interval of 1 minute) and once in the second session that took place 24 h after the first one (Figure 3). According to the definitions of Oliver and Rushton [24], PO was defined as* “the moment when the least experience of pain was recognized”* and SP as* “the moment when pain or tingling increased and the subject wanted the test to cease.”* Randomization of the side to test was initiated by asking the first participant to choose between two cards hidden in an envelope. The next participant was allocated in the opposite order and the same method was used for all subsequent participants. During the ULNT1 execution, gleno-humeral movements were not to exceed more than 10° (arbitrary set) of deviation from the starting position. If so, participants were excluded from the study. Participants were also excluded if SP was not elicited throughout the full available range of motion.

*Participants' Instruction and Experimental Set Up*. A standard explanation of PO and SP definitions was given and a demonstration of the ULNT1 was performed on the nontested arm by the operator. Participants were instructed to stop the ULNT1 at PO and SP. They laid supine with legs straight and both hands on their belly. A soft, thin (4 cm) foam pad was positioned under the head to avoid discomfort. The brackets of the positioning device were placed over the acromion-clavicular joints, bilaterally, as shown in Figure 2. The pressure unit mounted on the bracket of the tested side was inflated at a base level of 40 mmHg, and a cranio-caudal pressure was applied through the bracket until the pressure increased to 42 mmHg. The opposite shoulder girdle was maintained in a neutral position. The starting position (90° of glenohumeral abduction, 90° of elbow flexion) (Figure 3) was reached using the set square as a reference, by placing its nook on the jugular sulcus, and the two branches on the umbilicus and the medial elbow epicondyle, respectively. Once the starting position was reached, an offset of the goniometer was performed.

*Data Acquisition*. During each ULNT1 performance, participants indicated the occurrence of PO and SP by pressing the switch button and completed the VAS after SP was reached. The AbOS was calculated for each of the three ULTN1 performances. AbOS1, AbOS2, and AbOS3 corresponded to the angle measured during the first, second, and third ULNT1, respectively (Figure 3).

### 2.5. Statistical Analysis

Descriptive statistics were used to present data. The distribution of AbOS, PO, and SP occurrence and VAS values were tested using the Shapiro-Wilk's test (*α* = 5%).

The intrasession test-retest reliability of the AbOS was examined by considering AbOS1 and AbOS2, while the intersession reliability by considering AbOS1 and AbOS3. The test-retest reliability was examined using the Intraclass Correlation Coefficient (ICC) and Bland-Altman plots (B-A plots) [34] as recommended in studies on reliability [31, 34–36].

The ICC ranges from 0 to 1, with values closer to 1 representing the higher reliability and 1 meaning that “reliability is perfect” [31, 35]. The criteria used for the interpretation of the ICC in this study were as follows: 0.00–0.25: no correlation; 0.26–0.49: low correlation; 0.50–0.69: moderate correlation; 0.70–0.89: high correlation; and 0.90–1.00: very high correlation [37]. ICC “model 3, form 1” was adopted [38, 39].

B-A plots (CI: 95% and limits of agreement set at ±1.96 standard deviations) were generated to provide the limits of agreement and the visual representation of the size and range of differences between two AbOS from the same participant. The same statistical analysis was performed to test the intra- and intersession reliability of the angles of occurrence of PO and SP and pain intensity at the moment of SP.

IBM® SPSS® Statistics 20.0.0 (IBM, Segrate, Milano, Italy) was used to run statistical analysis. Significance level was set to *p* < 0.05.

## 3. Results

From the initial sample of 44 participants, two were excluded due to equipment failure (wi-fi connection failure between the hub and the G3 antenna) and 13 because the deviation of gleno-humeral movements exceeded 10° from the starting position. The final sample consisted of 29 participants.

The measurements collected were not normally distributed, as assessed by the Shapiro-Wilk's test (*p* < 0.05). The median and minimum and maximum values of AbOS1, AbOS2, and AbOS3 and the angles of occurrence of PO and SP, and VAS are presented in Table 1. 

**Figure 4:**
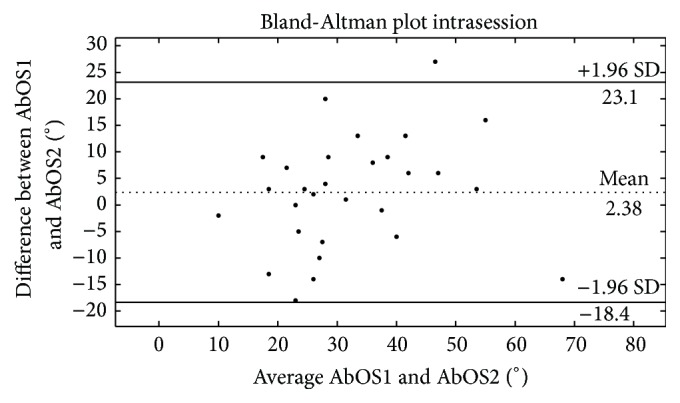
Bland-Altman plots showing the intrasession test–retest reliability of the angle between PO and SP (AbOS). The difference between two consecutive AbOSs is plotted against the mean of the same AbOSs. The central dotted line shows the mean of the differences. The two lines above and below the mean represent the 95% upper and lower limits (two standard deviations).

**Figure 5:**
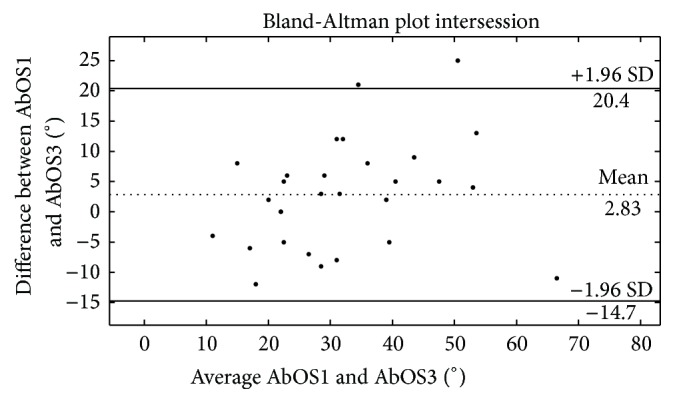
Bland-Altman plots showing the intersession test-retest reliability of the angle between PO and SP (AbOS). The difference between two consecutive AbOSs is plotted against the mean of the same AbOSs. The central dotted line shows the mean of the differences. The two lines above and below the mean represent the 95% upper and lower limits (two standard deviations).

*AbOS*. The ICC values for AbOS1_AbOS2 and AbOS1_AbOS3 were 0.71 (95% CI: 0.47; 0.85) and 0.79 (95% CI: 0.60; 0.89), respectively. The mean difference AbOS1 − AbOS2 as calculated in the B-A plots was 2.3° and the 95% limits of agreement were −18.3° and 23.1° (Figure 4). The mean difference AbOS1 − AbOS3 was 2.8° and the 95% limits of agreement were −14.7° and 20.4° (Figure 5). 

*PO Occurrence Angle*. The ICC values for PO1_PO2 and for PO1_PO3 were 0.46 (95% CI: 0.11; 0.70) and 0.64 (95% CI: 0.37; 0.81), respectively. The mean difference PO1 − PO2 as calculated in the B-A plots was 2.5° and the 95% limits of agreement were −33.9° and 28.8°. The mean difference PO1 − PO3 was 5.3° and the 95% limits of agreement were −30.7° and 19.9°.

*SP Occurrence Angle*. The ICC values for SP1_SP2 and for SP1_SP3 were 0.55 (95% CI: 0.23; 0.76) and 0.48 (95% CI: 0.15; 0.72), respectively. The mean difference SP1 − SP2 as calculated in the B-A plots was −0.1° and the 95% limits of agreement were −26.8° and 26.5°. The mean difference SP1 − SP3 was −2.5° and the 95% limits of agreement were −29.2° and 24.1°.

*VAS Recorded at SP*. The ICC values for VAS1_VAS2 and for VAS1_VAS3 were 0.92 (95% CI: 0.83; 0.96) and 0.87 (95% CI: 0.75; 0.94) respectively. The mean difference VAS1 − VAS2 as calculated in the B-A plots was −0.4° and the 95% limits of agreement were −2.0° and 1.1°. The mean difference VAS1 − VAS3 was −0.1° and the 95% limits of agreement were −2.0° and 1.7°.

ICC and B-A plot values are reported in Table 2.

## 4. Discussion

The intra- and intersession reliability of the angle between PO and SP during the ULNT1 was investigated. Furthermore, the intra- and intersession reliability of PO and SP occurrence in the range was tested. The results showed a “high level” of reliability for the first measurement and a “low level” of reliability for the latter ones.

An experimental setting was chosen for this study. Custom devices for participants positioning and an electromagnetic goniometer were used to guarantee (as much as possible) an appropriate level of accuracy during ULNT1 performance and data acquisition. The time interval between two consecutive ULNT1 was set to 1 minute to maintain as stable as possible the participants' condition, as suggested in some recent studies with a similar experimental design [40, 41]. For the same reason, the time interval between two different data acquisition sessions was limited to 24 h. This was especially relevant when investigating the intersession reliability, since the complexity of participant positioning and ULNT1 performance might affect the reliability of PO and SP measurements [9]. To our knowledge this was the first attempt to specifically investigate the reliability of the angle between PO and SP during ULNT1.

The angle between PO and SP measured during each ULNT1 performance corresponded to approximately 30°; this was different from other studies on healthy individuals where PO occurred 10° earlier in the range than SP [9, 23]. A possible explanation for this difference is that the starting position included a 90° gleno-humeral abduction instead of the 110° used in those studies.

The ICC and B-A plots suggested a high level of reliability both for the intra- and the intersession reliability of the angle between PO and SP, with a slightly higher ICC and a smaller amplitude of B-A plot limits of agreement for the inter-session reliability. No previous data are available for this variable. Only Coppieters et al. [9], while examining the intersession reliability of PO and SP occurrence in the range, found a lower correlation between measurements in the intersession reliability, both for the PO and the SP angles.

The intrasession reliability of PO and SP occurrence in the range, as measured in this study, was substantially lower than in results of previous research that reported a high intrasession reliability [9, 23, 24, 27]. The ICC values were indicative of a low correlation for PO angles and a moderate correlation for SP angles, while previous studies reported a “high” to “very high” correlation for the same measurements in both conditions. Although the ICC results were lower, according to Vanti et al. [23] a slightly higher level of reliability was found for SP angles compared to PO ones. Despite the mean difference between two consecutive measurements of PO and SP angles being close to “0” the width of the 95% limits of agreement was too high and not acceptable for this measurement to be considered reliable. Several reasons for such a difference in the results compared to other studies can be argued. Vanti et al. [23] found that the intrasession reliability was slightly higher for more expert physiotherapists; the operator performing the ULNT1 in this study had limited expertise. With that being said, the high intra- and intersession reliability of the VAS measurements recorded at SP suggests that the ULNT1 was performed properly. Speed changes in neurodynamic test performance can induce different pain responses [42]; although the operator tried to perform the ULNT1 at the same speed with all participants, differently from other studies [27], no accurate control of the elbow extension speed was carried out. Finally, the cut-off adopted as an exclusion criterion (not more than 10° of gleno-humeral movement from the starting position) could have been excessively wide.

Interestingly, considering all these limiting factors, the intra- and intersession reliability of the angle between PO and SP was high.

## 5. Conclusions

Besides the commonly proposed criteria for a positive neurodynamic test, other variables may be considered to quantify neural mechanosensitivity. For the first time to our knowledge, the intra- and intersession reliability of the angle between PO and SP during the ULNT1 was specifically investigated and compared to the intra- and intersession reliability of PO and SP occurrence in the range. Measurement of the angle between PO and SP was more reliable than the measurement of PO and SP angles of occurrence in the range. Results were indicative of a high intrasession and a high intersession reliability. Taking this into account, the measurement of the angle between PO and SP may be a suitable (more reliable) alternative for the assessment of neural mechanosensitivity. Further research should focus on the reliability of this measurement in a clinical setting.

## Figures and Tables

**Table 1:** Descriptive statistics.

	Angle between PO and SP (°)			PO occurrence in range (°)			SP occurrence in range (°)			Pain intensity at SP (1–10)		
	AbOS1	AbOS2	AbOS3	PO1	PO2	PO3	SP1	SP2	SP3	VAS1	VAS2	VAS3
Median	32	31	27	38	43	43	71	72	73	7	8	7
Range	9; 63	11; 75	11; 72	1; 64	2; 60	1; 60	19; 96	39; 98	50; 96	1; 9	1; 9	1; 9

**Table 2:** Intraclass correlation coefficients (ICC) and Bland-Altman plots (B-A plots) values for the angle between PO and SP (AbOS) and the angle of occurrence in range of PO and SP.

	ICC [upper; lower bounds]			B-A plots mean difference [95% limits of agreement]		
	AbOS	Pain onset	Submaximal pain	AbOS (°)	Pain onset (°)	Submaximal pain (°)
Intrasession	0.71 [0.47; 0.85]	0.46 [0.11; 0.70]	0.55 [0.23; 0.76]	2.3 [−18.3; 23.1]	2,5 [−33.9; 28.8]	0,1 [−26.8; 26.5]
Intersession	0.79 [0.60; 0.89]	0.64 [0.37; 0.81]	0.48 [0.15; 0.72]	2.8 [−14.7; 20.4]	5,3 [−30.7; 19.9]	−2.5 [−29.2; 24.1]
